# Emergent Dynamics from Spiking Neuron Networks through Symmetry Breaking of Connectivity

**DOI:** 10.1371/journal.pone.0064339

**Published:** 2013-05-17

**Authors:** M. Marmaduke Woodman, Viktor K. Jirsa

**Affiliations:** Institut de Neurosciences des Systèmes Inserm UMR1106 Aix-Marseille Université, Marseille, France; University of Maribor, Slovenia

## Abstract

Low-dimensional attractive manifolds with flows prescribing the evolution of state variables are commonly used to capture the lawful behavior of behavioral and cognitive variables. Neural network dynamics underlie many of the mechanistic explanations of function and demonstrate the existence of such low-dimensional attractive manifolds. In this study, we focus on exploring the network mechanisms due to asymmetric couplings giving rise to the emergence of arbitrary flows in low dimensional spaces. Here we use a spiking neural network model, specifically the theta neuron model and simple synaptic dynamics, to show how a qualitatively identical set of basic behaviors arises from different combinations of couplings with broken symmetry, in fluctuations of both firing rate and spike timing. We further demonstrate how such network dynamics can be combined to create more complex processes. These results suggest that 1) asymmetric coupling is not always a variance to be averaged over, 2) different networks may produce the same dynamics by different dynamical routes and 3) complex dynamics may be formed by simpler dynamics through a combination of couplings.

## Introduction

The mechanistic explanations employed in behavioral, cognitive and neural sciences often take the form of network models and their dynamics. Various signatures of nonlinear dynamical phenomena are ubiquitous in these disciplines, e.g. phase transitions, pattern formation and time-scale separation. Yet, in the literature, one does not find a systematic account of the relationship between the emergence of the dynamics of behavioral and cognitive processes and the dynamics of the underlying neural networks and their structural properties. We argue here that such an account of the structure-function relationship begins by understanding the different ways the structure of a neural network leads to its collective dynamics. In particular, we focus on the contributions of network connectivity as a means to control the emergence of arbitrary low-dimensional dynamics.

The nonlinear nature of human perception and action dynamics is well documented, with the early example of the Necker cube. In general, hysteresis and autonomous switching in cases of perceptual ambiguity have been modeled in terms of a bistable system [1]. Elsewhere, critical fluctuations and entropy increase have been measured in the performance of cognitive tasks [2]. Flows of state variables have been reconstructed systematically for movements in various task paradigms [3]–[6]. These flows are typically defined in two- or three dimensional spaces and comprise invariant elements including stable fixed points, saddle points, limit cycles, stable manifolds and separatrices. It has been shown that functional relevance can be attributed to the various flow features. For instance, a separatrix in state space may act as a threshold element and can be measured through perturbation studies [7]. As task demands change, for instance through changes of task difficulty [6], then the flow of the behavioral dynamics changes as a whole and undergoes bifurcations. These findings demonstrate that the entire flow of the emergent collective dynamics plays a functionally relevant role and a description of only some of its components (such as fixed points) offers only a partial understanding.

Recent examples from neuroscience are available on the temporally extended neural processes underlying such behaviors. For examples, Graziano and colleagues stimulate a local (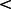
1 mm) part of neural tissue. Only long (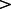
200 ms) durations can trigger temporally extended movement trajectories by inciting spreading and sustained activation of motor networks [8], [9]. Related work by [10] shows that spiking activity of certain neurons in the motor cortex is better predicted by movement trajectory rather than position. Studies employing tasks with more behavioral structure find, for example, that supplementary motor area blood flow is linked to stability of rhythmic coordination [11], and reorganization of electroencephalogram activity is linked to transitions in rhythmic coordination [12]. It has also been shown that rhythmic neural activity in the delta and beta bands of human local field potentials may track task structure and completion [13], and motor neurons show spike correlations in anticipation of stimulus presentation [14]. Such evidence suggests a strong tendency of neural activity to organize in temporally extended, neurally distributed processes relevant to behavioral function.

**Figure 1:**
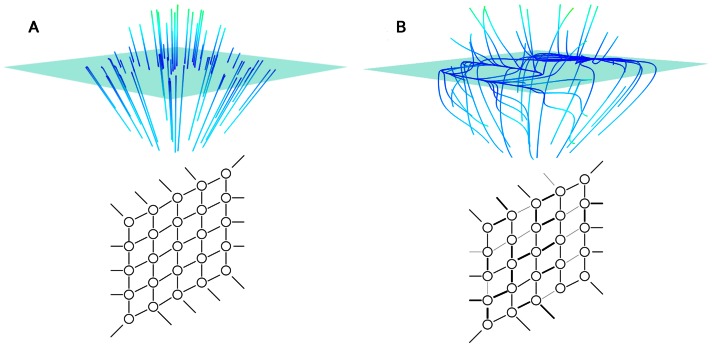
Phase flows and manifolds generated by high dimensional systems. Diagrammed on the left and right are cases of a fully symmetric system and one with asymmetries. On both sides, the top is a cartoon of the dynamics and the bottom shows a diagram of a network and its connections. **A** In the symmetric system, where all connections are the same, the phase space contains an attractive manifold (the cyan surface), and all trajectories collapse to fixed points on the manifold (blue lines). **B** In the asymmetric system, when the system is away from the manifold, the dynamics are dominated by symmetries, and the trajectories collapse toward the manifold, but as they approach, the asymmetries in the network begin to be expressed, producing slow flows on the manifold. Here, the manifold has a limit cycle on one side of a separatrix, and a single fixed point on the other side.

Previous modeling work has studied neural network dynamics and interpreted such models in the context of behavior. Prominent examples are found in the domain of decision making [15], [16] and movement coordination [17]. General theoretical results exist on manipulations of network parameters, typically resulting in some form of symmetry breaking in its widest sense, but almost exclusively focus on the (de-)stabilization of a chosen attractor such as a fixed point or the synchronization of coupled oscillators. Kuramoto studied the synchronization of weakly coupled oscillators and dispersed eigenfrequencies in his seminal work [18]. Multistability and synchronized assemblies may arise from homogeneously coupled networks as a consequence of their interaction function for strong couplings [19] and weak couplings [20]. The use of phase response curves allows analytical insights into the stability of coherent network activity [21], [22]. Even in absence of all coupling, a set of oscillators with a common stochastic drive may display synchronized clusters depending on the form of their individual oscillator dynamics (more precisely the asymmetry of the flow in state space as expressed by the nonlinearity) [23]. In these examples the symmetry breaking does account for emergent dynamics, generating different modes of activity across the network through the asymmetry of individual oscillator parameters. Recent work by Rabinovich and colleagues [24] have begun to demonstrate the extent to which asymmetries in the connectivity may reshape the emergent dynamics of a network of three neurons. These authors considered specifically the change from a multistable attractor to a limit cycle. In the current work, we wish to advance this view by showing how arbitrary flows in state space recur where symmetries in coupling are broken in general spiking neural network models, as illustrated in Fig. 1. We will examine the formation of manifolds and phase flows in the dynamics of firing rate and spike timing. Such principles also predict that in more complex behaviors, where low dimensional attractors are only transiently stabilized by dynamics on slower timescales, that transitions between fundamental spatiotemporal patterns will be found by examining changes in the effective or ‘working’ subspace of the network's entire state space.

## Results

In the following we will discuss results from firing rate and spike timing representations of networks in various degrees of complexity to elucidate the principles of functional emergence from structural motifs. Specifically, we study the dynamics of a spiking neural network comprising the theta neuron model and synaptic couplings as shown below (see the methods section for details):
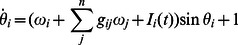
(1)

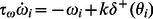
(2)where the state variables 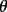 and 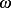 correspond to spiking and rate variables, 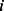 and 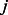 index the post- and pre-synaptic neuron, 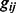 is the coupling value from neuron 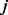 to 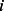, and 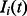 is the non-autonomous input to neuron 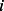. 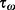 and 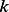 are the timescale of 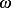 dynamics and the strength of the effect of a spike on 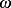, where the values are typically set to 20 ms and 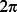, respectively.

In the following we shall first consider the slow dynamics of the firing rate in Equation 2, followed by an examination of its spike timing dynamics.

### Firing rate

#### Mode formation

**Figure 2:**
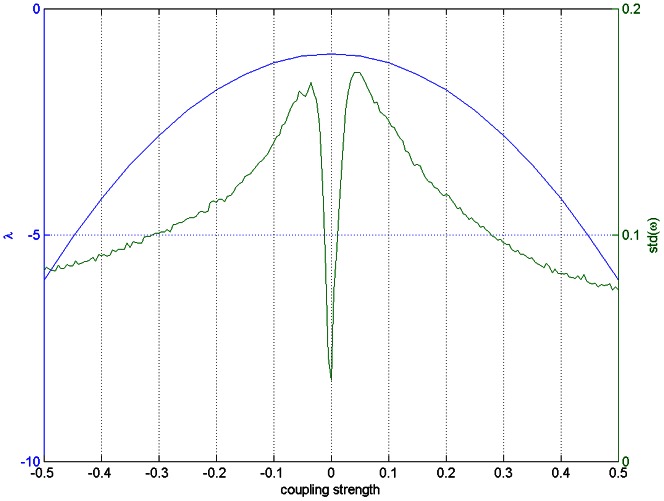
Rate mode formation. In blue is plotted values of the eigenvalue determining the stability of a mode in rate dynamics under the assumption of no input and homogeneous coupling, where the green line shows the standard deviation of 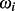 steady state values for simulations with given global coupling values.

One of the first questions that can be posed about the relation among components in a system is whether they coordinate. Given the reduction of network dynamics to its firing rate 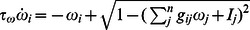 of our neural network, as developed in the methods section, we study the effect of the symmetries 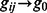 and 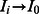, which give
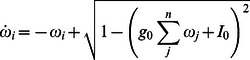
where the timescale factor of 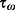 has been dropped for simplicity,
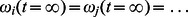
where all nodes have the same steady state dynamics, coupled to the mean field. They thus all have the same nullcline equation and must all be equal, satisfying the equation
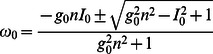
where we consider the positive solution only. To determine the stability, the eigenvalues of the corresponding Jacobian are computed: there are 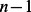 eigenvalues of 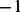 and a single unique eigenvalue determining stability
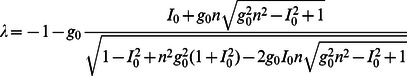
We show the stability of the fixed point in Fig. 2 where the mode is more stable as the coupling value increases: 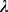 becomes more strongly negative in both directions away from zero coupling, and in simulations of global homogeneous coupling with normally distributed constant inputs 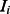, the standard deviation of the steady state values of 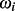 decreases away from zero coupling, demonstrating that strong and homogeneous coupling values lead to the formation of modes in firing rate dynamics under condition of inhomogeneous input.

The above result of symmetric coupling is interpreted simultaneously as generating a single fixed point attractor in the phase space of the network but also as generating a corresponding functional mode. The latter functional mode 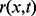 can be decomposed as a function of time and space via the relation 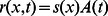 where 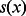 describes the spatial pattern and 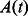 describes the time dependent amplitude of the mode. We note that the formation of such modes results from symmetry in coupling and intrinsic dynamics of the system.

#### Phase flows

Where symmetries generate the functional modes in a network, resulting in fast contracting dynamics towards attractors in the phase space, the symmetry may be broken as well in the coupling or component dynamics. Where multiple modes exist in the same system, symmetry breaking generates slow dynamics in the phase space spanned by these modes, what we refer to as a phase flow. In the following, we will start by examining the phase flows generated by symmetry breaking in two mode networks by examining two neuron networks, and we will afterward consider phase flows of three modes. We refer to the set of phase flows that are seen below as Excitator dynamics, on which we elaborate in the methods section. Throughout, we wish it be understood that while we shall demonstrate the phase flows in terms of networks with a few neurons, the phase flows can be equally generated by networks, which possess several stable modes.

**Figure 3:**
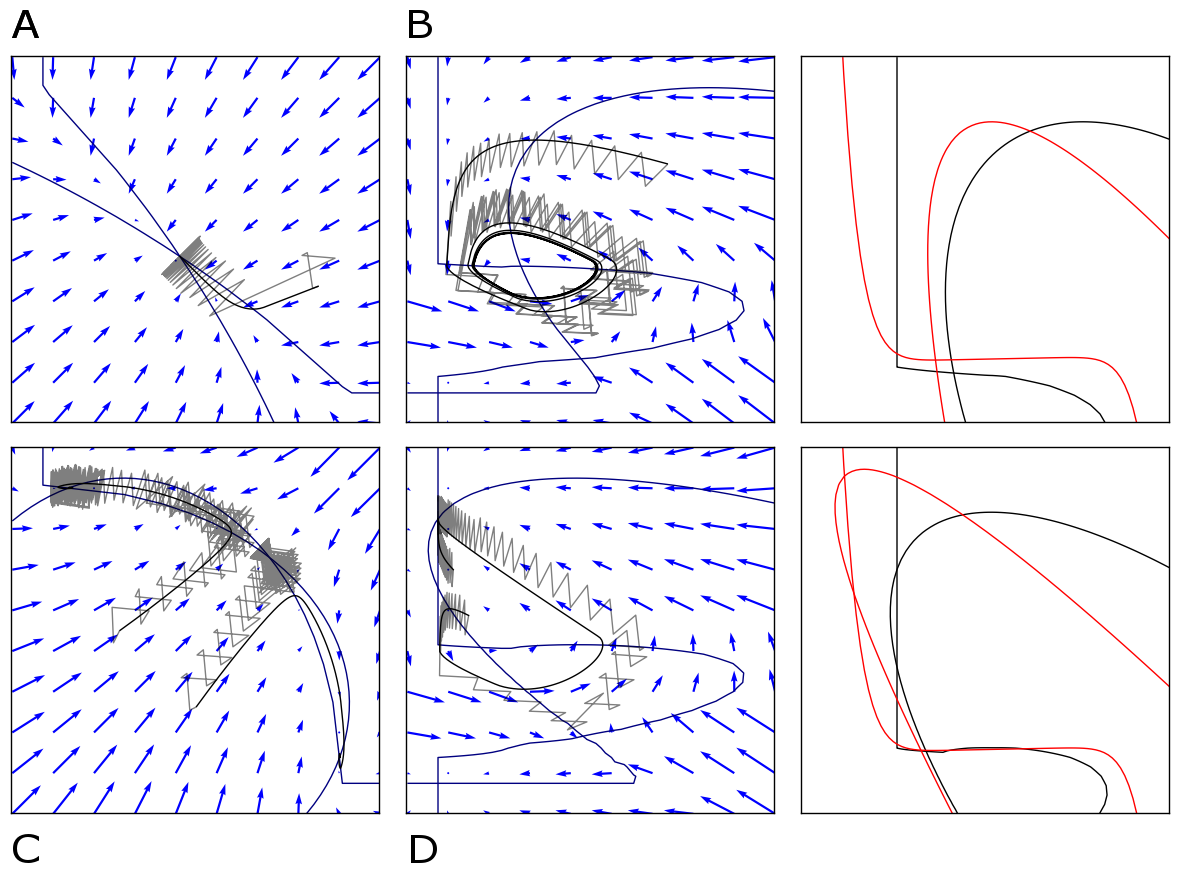
2D network rate dynamics. Demonstrated here are four kinds of dynamics generated by a two neuron network. In the left two columns, the vector field is shown in blue, and the dark blue lines show the nullclines. Trajectories from simulations of rate equations are shown in black while trajectories from simulation of the corresponding spiking network are shown in grey. **A** fixed point, **B** limit cycle, **C** bistability, and **D** monostability. In the right column, a subset of the phase space is shown as structured by the nullclines of the rate dynamics in black, with polynomial approximations of those nullclines overlayed in red.

For illustration, we consider a network of two nodes, where the connectivity matrices of generating the phase flows are shown in Fig. 3. Each matrix is considered both in terms of the coupling values themselves and a decomposition
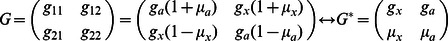
where 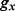, 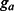, 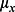 and 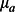 are the connectivity strength between neurons, strength of self-coupling, difference in strength between neurons and difference between self-coupling, respectively. Each connectivity matrix is thus decomposed into its symmetric and asymmetric or antisymmetric components. In the table 1, we summarize the connectivity matrices generating each of the phase flows in the panels of Fig. 3. The phase flows of Fig. 3 (see the two left columns) nicely demonstrate the emergent topological features as expected from the Excitator dynamics. The nonlinearities in these systems are strong as can be appreciated from the shapes of the nullclines in Fig. 3. In principle it is possible to fit polynomial expressions to these flows and hence derive the differential equations in the more familiar form of polynomial couplings. We demonstrate this and the polynomial representation as proof of concept in the right column of Fig. 3, where we overlay polynomial approximations of the nullclines of the dynamics (red curves) with the real nullclines (black curves) located in the part of the phase space with the topological features we are interested in. It is evident that both representations show the same topological features and therefore result in phase flows that are qualitatively identical.

**Table 1:** Coupling used in the two dimensional networks.

Attractor type	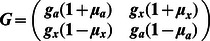	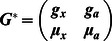
Fixed point	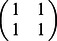	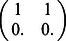
Limit cycle	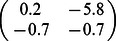	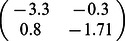
Bistability	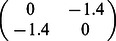	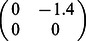
Monostability	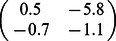	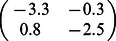

We give examples here of coupling matrices sufficient to produce the varied dynamics shown in Figure 3.

**Figure 4:**
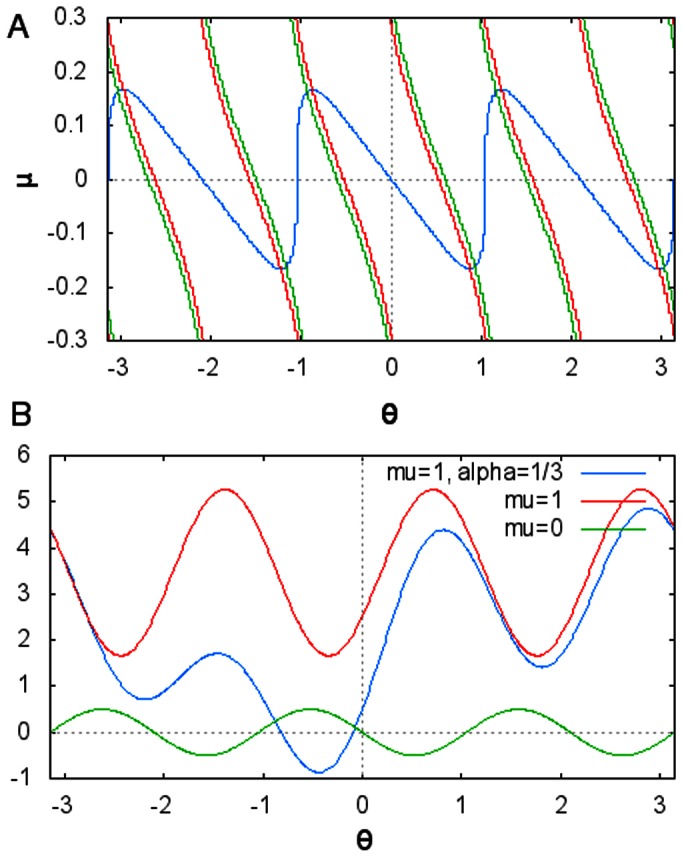
3D network analysis. **A** The fixed points of the derived phase equation (blue) as a function of asymmetry strength 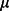. Above certain values, no fixed point exists, system bifurcates to limit cycle. **B** 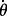 as a function of 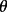. Different degrees of asymmetry in the network connectivity yield different phase flows. In green, connectivity is symmetric, and the system has three multistable fixed points. As 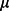 increases to 1 (red), a bifurcation to limit cycle occurs. A further asymmetry is introduced by 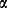 (blue) which produces a monostable dynamics with one fixed point and a nearby separatrix.

In networks of three neurons or neural modes, we first consider one three-way asymmetry in a fully coupled network, as described by the connectivity matrix
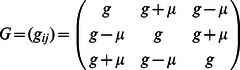
where 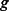 is again the strength and 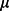 is the degree of asymmetry. Such a network is the most basic example of a heteroclinic cycle [24], and the activity can be cyclic if 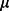 is high enough. In order to identify the parameter regime producing cycle behavior, we drop the square root in Eq 15 and set 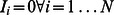 to obtain
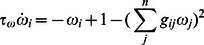
(3)in order to simplify the analytics that follow, yet in the relevant parameter regime, this system produces the same qualitative dynamics. We reduce this network with the above asymmetries
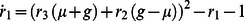


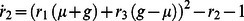


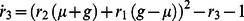
to a phase equation to show the bifurcation from multistability to limit cycle. We obtain the necessary projection by using the right singular vectors of the asymmetry matrix,
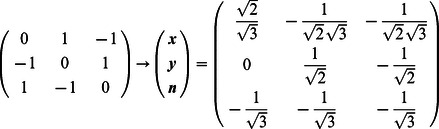
as the normal, 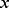 and 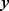 components of the vector field, and then using a polar transform of 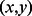 to 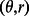, we obtain
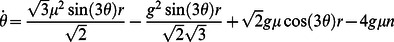


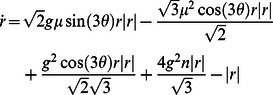


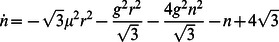
where we then average the radius and normal dynamics over phase to find 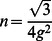 and 
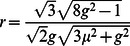
. Substituting these into the phase equation, we have
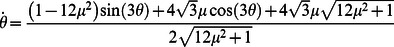
(4)where we have used 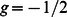 as an example to simplify this equation. We note that this equation assumes that the system stays near the manifold corresponding to the average radius and normal dynamics. In Fig. 4 we show how solutions vary with asymmetry.

We introduce a further asymmetry in the matrix used above
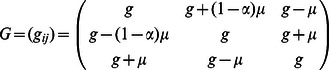
and apply the same projection, average normal and radius dynamics and obtain a phase equation 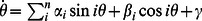 where 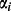, 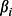 and 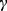 are sixth order polynomials in terms of 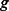, 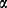 and 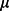. The full equation is too complex to be of use without further simplifications or reductions, but in Fig. 4, the phase portrait of the system is shown for different sets of asymmetries.

While the above results were obtained with a modified reduced network equation, the results remain useful for understanding the full spiking network. In Fig. S1, we show simulations of the full spiking neuron network using the parameters found by the above analysis, and we find the same set of Excitator dynamics in the spiking network as predicted by the phase reduction of the rate equations.

### Spike timing

#### Synchronization

The network model being considered was also motivated by a desire to study, in the same model, the presence of dynamics of spike timing. We thus employ and analyze a phase reduction of the model in section
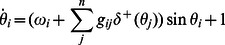
(5)where we have assumed that the averaged firing rate has been absorbed into the intrinsic firing rate term 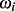, and that the pulses from presynaptic neurons can be idealized as Dirac delta functions (we have used 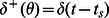 where 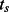 is the time of a spike defined by 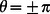).

**Figure 5:**
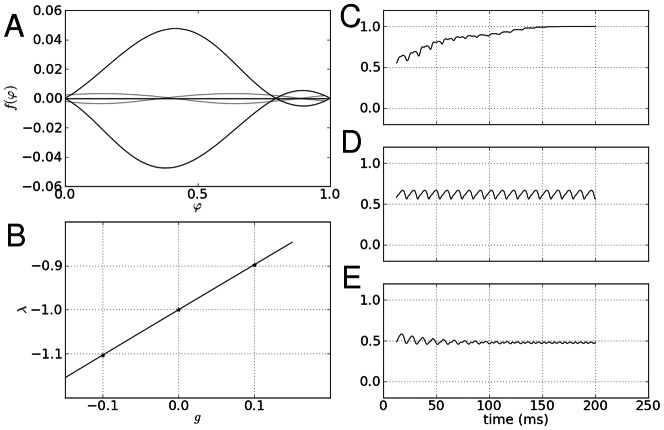
Phase mode stability with respect to phase and coupling strength in a symmetric network. **A** The phase response curve for three values of coupling strength which are indicated by stars in **B** where the stability of the mode is plotted with respect to coupling strength. In order for the mode to be stable, 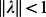 must be satisfied, so here we note that as coupling strength crosses zero to positive, the mode becomes stable **C, D, E** From top to bottom, time series of the mean field 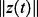 of simulated networks are shown, corresponding to the coupling values starred in the left, bottom panel. **C** the order parameter approaches one, due to a positive coupling value, and in **D**, where no coupling 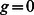 is present, the field exhibits oscillations in its mean field. In **E**, where coupling is negative, the phases become evenly dispersed.

In a network of phase oscillators, we approach synchronization, i.e. the formation of synchronous modes, by considering the Eq 5 with symmetric frequencies 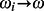 and coupling strengths 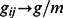, with 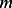 being the number of oscillators, and introducing a complex mean field 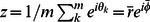, also known as the Kuramoto [25] order parameter,
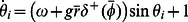
(6)For the purposes of deriving the stability of a mode, we assume that the steady state is one in which all oscillators are synchronized, i.e. 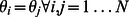, and we can, using Eq 17 write the general condition
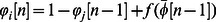
(7)which after perturbation and subtraction of the corresponding steady state becomes
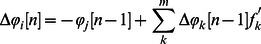
(8)The only necessity to solve this linear system is that we have 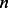 equations, so without loss of generality, we let 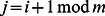, and then the matrix form is
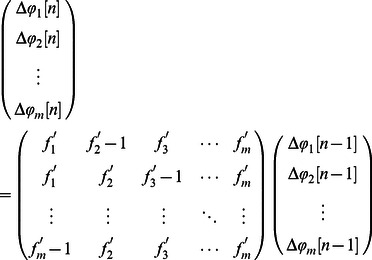
(9)which has 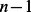 neutral eigenvalues and a single unique eigenvalue
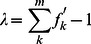
(10)The synchronous mode is thus stable if 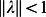. Because the phase equation, and thus 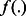, was formulated with a mean field, all 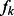 are identical, and we have
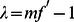
which is shown as a function of coupling strength in Fig. 5, as applied to Eq 18, the phase response curve 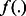 derived for this theta oscillator.

As in the case of dynamics of firing rate, mode formation in spike timing corresponds to fixed points in the relation between spike timing, yet breaking the symmetry between neurons in the network can lead to a time scale separation through which a slow dynamics is generated.

#### Relative phase dynamics

**Figure 6:**
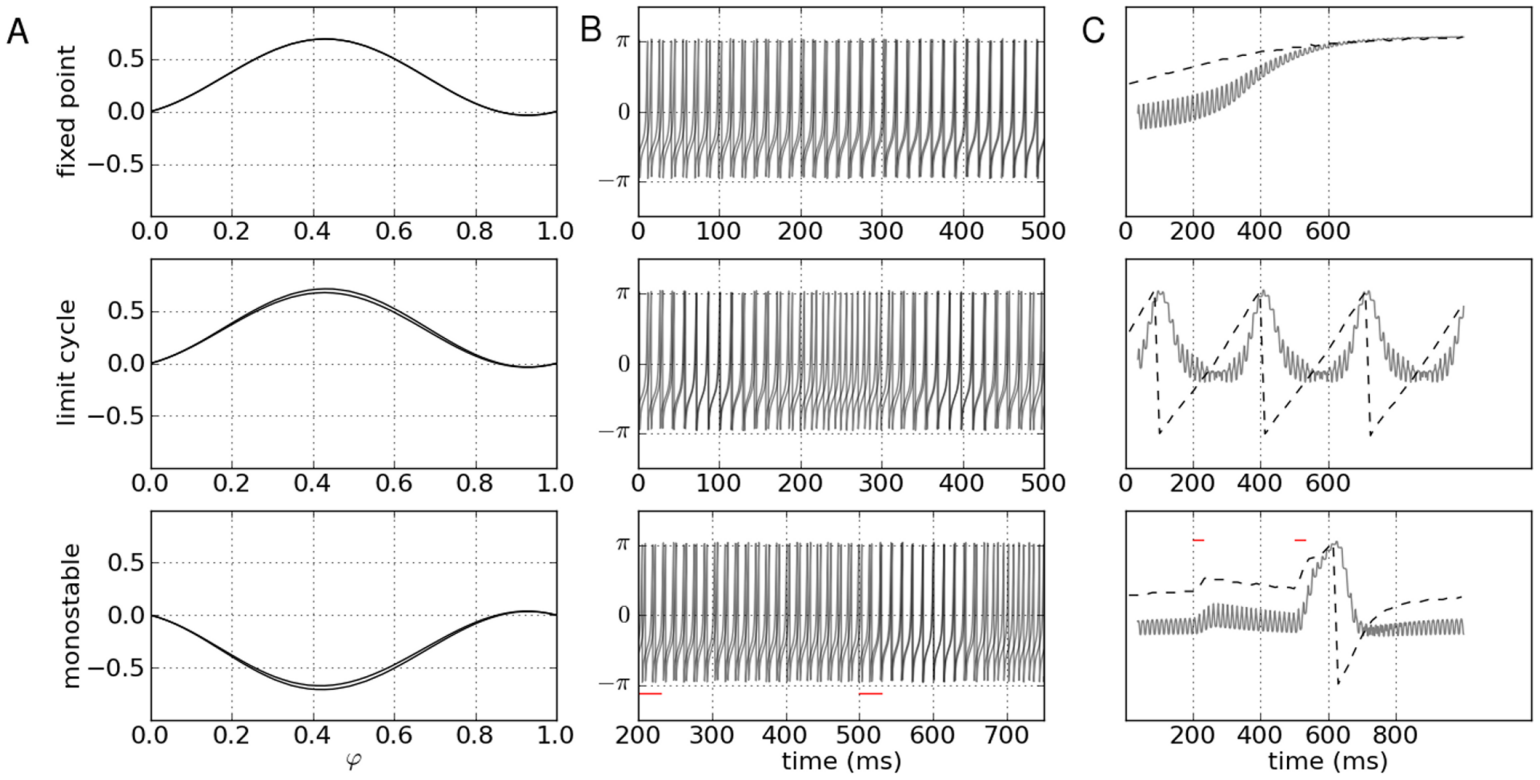
Spike timing flows. Presented here are different possible dynamics of spike timing in a two dimensional network. The top, middle and bottom rows correspond to fixed point dynamics, or synchrony, limit cycle or asynchrony, and monostability, where there is a weakly stable fixed point, which can be perturbed to produced transient trajectories (red dashes). **A**: phase response curves of the two neurons in the network, normalized by the magnitude of coupling strength. **B**: time series of the spiking dynamics 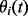 **C**: The mean field 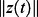 is shown in gray and the relative spike timing modulo the period is shown by the dashed line. Both indicators of phase organization show the distinct qualitative differences in dynamics between the three phase flows.

Such phase flows for spike timing dynamics take the form of flows in the relative phase of the oscillators, which depend on the asymmetries between neuron or modes in the network. In Fig. 6, we demonstrate the existence of Excitator style dynamics in the spike timing of a two neuron network. When the neural responses are identical and the synchronous solution is stable, the relative phase between the two neurons converges to zero. When the synchronous solution becomes unstable due to slight symmetry breaking in the coupling, no fixed value is observed for the relative phase, but rather an oscillation of the relative phase emerges on a slow timescale. The third case we show is that of monostability, which arises also from asymmetries between the neurons: there exists one fixed point with a separatrix nearby such that small perturbations do not disrupt the antiphase firing pattern while a strong enough perturbation produces a transient synchronization. Not shown here are simulations demonstrating multistability; such dynamics are observed as multistable configurations of firing order, requiring at least three dimensions, and have been investigated previously by [26]. The dynamics of such relative phase organization are also reflected in the mean field of the networks.

### Composition of dynamics via composition of connectivity

**Figure 7:**
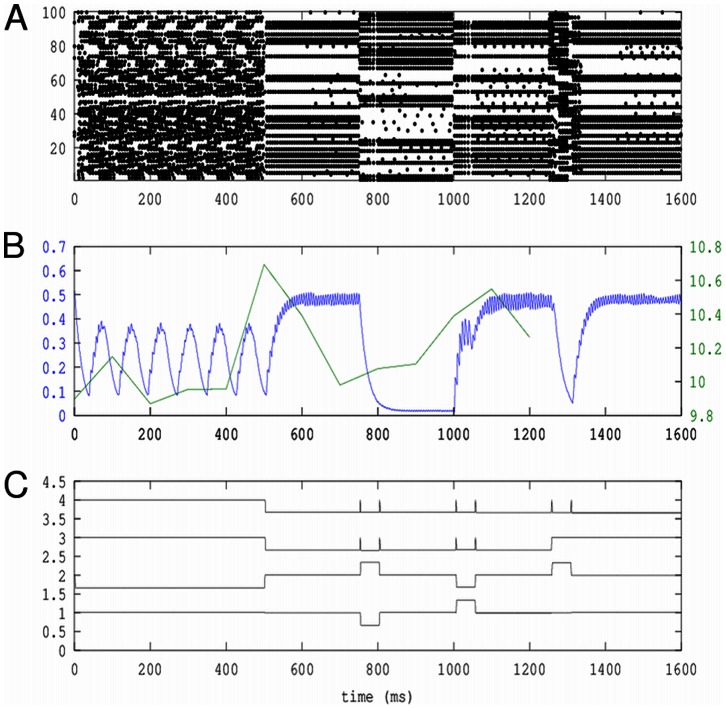
Multifunctional circuit. Here shown are characteristic time series of a multifunctional neural circuit, which in time, is switched from a limit cycle flow, to bistability, where perturbations flip the active mode back and then forth, and lastly monostability, where a perturbation induces a transient. **A**: Raster plot of spike times of the network. **B**: Time series of the first principal component of 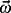, corresponding to the network which takes part in each of the phase flows, shown in blue. In green is shown a sliding measure of subspace similarity (see text for details), which takes its greatest values when the phase flow produced by the network switches. *C*: Time series of the input pattern applied to the network, including perturbations, simulating the effect of control signal signals on a longer timescale.

While the results above show how the most basic classes of behaviors arise in network dynamics, we set out to show that complex nonlinear processes can be composed of more basic ones. First, the dynamics shown above are demonstrations in networks with two or three neurons, yet in the case of pattern formation, such dynamics is distributed across a network. Second, complex processes involve temporal composition, in which a set of basic elements are given a sequential structure. To better understand how such aspects would play out in more complex scenarios, we constructed a larger network, composed of four neural *modes*, which combines three of the phase flows show in Fig. S1, namely bistability, monostability and limit cycle, using the following connectivity skeleton
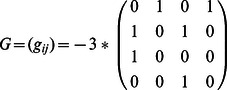
which is then distributed across twenty five spiking neurons. Modes 1 and 2 produce bistability; 1, 2 and 3 produce monostability, and 1, 3, and 4 produce a limit cycle. As shown in Fig. 7, we simulate the effect of a longer timescale control signals by using per mode input patterns are used to inhibit those modes that do not produce a particular phase flow, thus reshaping dynamically the effective phase space: first, a limit cycle flow is chosen, then, at 500 ms, the input inhibition pattern selects the bistable phase flow. During the production of bistability, we perturb the network with additional input to switch the dominating mode first at 700 ms, and again at 1000 ms. At 1100 ms, the network is switched to a monostable pattern, which appears first as a fixed point, until a perturbation induces a large transient activation.

In the figure, we have shown in the top panel a raster plot of the spiking activity of the network. The spatial patterns corresponding to the modal distribution in the network are clearest during the bistable and monostable flows but can be discerned during the limit cycle as well. In the middle row in blue, we have shown the mean field activity of the first mode, which participates in all three phase flows; this time series is identical to that of the first component from a principal component analysis of the synaptic currents induced by the spiking activity (as approximated by 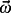). We then attempted to estimate the transitions in phase flow by performing a sliding window principal component analysis, using a window length of 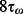 and an overlap of 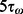, corresponding to 160 and 100 ms, respectively, resulting in a series of principal eigenvector sets, 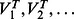, each of which characterizes the phase space in terms of principal variance. In order to capture how the active subset of the network's phase space changed over time, for every pair of set of eigenvectors 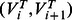, we computed the similarity as the sum squared difference between 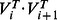 and the identity matrix, for the first ten principal eigenvectors. This measure is plotted over time in green, and rises as the network goes through phase transitions and falls between them. Critically, this suggests that transition in the dynamics of modes can be detected by examining the effective subspace of the network's phase space. In the bottom panel, we have plotted the input patterns applied to the network modes, as function of time.

## Discussion

Our goal is to demonstrate how arbitrary low-dimensional flows may systematically emerge from networks due to the breaking of symmetry in couplings. A broader scheme would consider symmetry breaking in a wider sense and incorporate also asymmetry in the intrinsic neuron dynamics. The key concept to our approach is that these patterns code for dynamical processes (structured flows on manifolds (SFM)), which themselves can be dynamically complex, nonlinear, multistable and display all the features of behavior known from cognitive sciences. Perdikis and colleagues proposed such SFMs as building blocks for cognitive architectures [27], [28]. The dichotomy of rate versus spike coding (c.f. Christof Koch [29] for a discussion of this subject) does not exist here, since the time series of the spiking neurons follow a systematic but not necessarily stationary behavior, and it is their ensemble dynamics (as opposed to ensemble state) that is the true carrier of the representation of the lower-dimensional functional process. The metrics applied to the time series as firing rates or spike timings can offer only an impoverished view by construction. These metrics often, nevertheless, make sense for certain functions, neural subsystems or even task conditions. For instance, Riehle et al. in [14] demonstrated that synchronization of individual spike discharges and its rate modulation can be involved differentially in certain motor cortical functions. In other motor task conditions Hatsopoulos [30] showed the appearance of non-stationary synchronization waves with precisely timed spiking in MI at a characteristic latency. The exact timing of this response varies depending on its relationship with ensemble activity as measured by the phase of the ongoing beta band oscillation. These findings clearly indicate that there is a role for both rate and spike code, but neither of them is necessarily independent. Furthermore, no relation to the behavioral time courses of the end effectors has been made. It is at this point where the notion of structured flows on manifolds, as developed here, will be able to conceptualize these findings within a common theoretical framework. Even though it may still be too early to delineate the detailed relations between spike timings, rate modulations and behavior for specific tasks, the principled behaviors of SFM-based functional architectures are nevertheless clear and predict timescale hierarchies, non-stationary but systematic dependencies between spike timings and spike rate, as well as systematic spiking behaviors of single neurons even for smaller firing rates. In the case of a rate-coding scheme, trajectories in the phase space of neuronal firing rates will inevitably traverse the lines or planes corresponding to integer ratios of firing rates, and a synchronous attractor will stabilize as the integer ratio is approached and destabilize as the firing rate ratio diverges from the integer value, thus producing observable transient synchronizations. This form of activity is important because it allows for neurally significant events to rise out of the mean field activity and more successfully signal downstream structures. It is thus expected that such transient synchronizations should track the structure of task dynamics, as well as coinciding with fluctuations in firing rate.

Two important forms of degeneracy are present in the network models in this work: First, for any given process a network may generate, the mapping of the generating dynamical mechanism onto a connectivity matrix may be achieved by many different structural configurations. This degeneracy is systematic and reflected mathematically by the adjoint mapping of 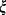 to 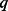. Second, there is an additional degeneracy with respect to the dynamical generating mechanism. We take for example the monostability displayed by the two and three dimensional rate flow networks and spike timing: all three networks could fulfill the same functional role, while the mathematical details of their implementation vary greatly as well as the underlying network structure. Such forms of degeneracy are ubiquitous in biological systems [31] and are though to contribute significantly to their adaptive functionality.

To account for the structure of more complex behaviors, it becomes necessary to understand how basic processes or primitives may be constituents of complex behaviors. In nonlinear or non-modular systems, compositionality is a nontrivial problem. The presupposition of timescale hierarchy allows for the decomposition of complex dynamics into simpler dynamics on multiple temporal scales. Such decomposition may either occur in parallel, which will give rise to two mutually coupled subsystems forming a hierarchy; alternatively the decomposition may occur sequentially (such as in the coexistence of slow and fast manifolds in the system dynamics), which will give rise to serial behaviors on different timescales (fast-slow). We showed the effectiveness of the former in the last simulation where control signals on a slow timescale reshape the effective phase space of the network; this reshaping produces produces transitions in the qualitative dynamics produced by the network that can then be identified by examining the changes in principal components over time. When applied in combination with sequential dynamics, phase space reshaping of SFMs may rapidly yield complex articulated processes, suggestive of how such processses may be structured in behavior.

Recent work [5], [27], [28] has used this notion of functional decomposition and presented a way of treating the dynamical phenomenology of human function as the fundamental object of study, employing hierarchical nonlinear dynamical systems as toy models and deducing necessary principles from them. The critical insight of these researchers has been that SFMs can be regarded as patterns of behavior. In other words, an SFM can be mathematically characterized as a pattern or mode, and as a consequence, a highly complex system can be decomposed into the set of behaviors, since the generating system dynamics can be decomposed into the set of corresponding SFMs. This approach has shown, in novel ways, how behavior in general may not only self-organize but self-adapt and self-compose via phase transitions and time scale hierarchies. Critically, in a dynamical model of handwriting [28] Perdikis et al show how phase flows may be systematically composed for the construction of complex function, by using certain dynamics to parametrize others, an example of which we have seen in this work, Fig. 7 where the phase space is dynamically reshaped.

Another formulation of how behavioral dynamics are embedded in neural networks has been extensively developed by Schöner and colleagues [32], within a framework termed Dynamic Field Theory. Said framework provides an interpretation of various formulations of Amari's neural fields [33], which, in the numerous applications from movement to psychophysics, have significant explanatory power by colocating representation of movement information and preparation in a dynamic neural field. The implication is that parameters of movement are defined in a continuous space, and that evolution of such parameters occurs continuously in time. It is important to note that such neural fields support a limited set of dynamics, mostly relying on the bistability of the equilibrium state and a local excitation, as well as variations thereof. Because the representation of task information rests in the activation of nonlinear dynamics of the neural field, Schöner et al suggest, in contrast to symbolic theories of cognition, that such representation, instead of being operated upon, performs its own operations. We note however that more complex autonomous dynamical structures, such as sequential dynamics, that require breaking assumptions of the Amari field model, e.g. homogeneity in the connectivity kernel, cannot be described without introducing a hierarchy of fields whereas in the current work, we have shown how a richer repertoire of dynamics may be accomplished with relatively simple network configurations.

The more general framework of liquid state computing, recently outlined in [34], presents another theory of how behavior may emerge from neural networks. A particular point which resonates with the present results is the dependence of emergent dynamics on the state of the network: we showed how the input pattern, which in the context of a larger model may consist of afferents from a higher order area, sculpts the dynamics of the network. We wish to draw a slight distinction between the results presented here and the theories of liquid state computing with respect to the emergence of function from neural structure. In our results, neurons' representational content may be specific to the phase flows in whose production they participate, while the theory of liquid state computing posits and indeed is founded upon the randomness of the network, in which function emerges through the projection of a high dimensional space onto a low dimensional space. Our results suggest a definite association between the subnetwork of neurons and the different function they may coordinate transiently in order to produce.

This has been clearly identified in the work of [14], where spiking events coincide in a statistically significant way, not necessarily in response to, but in anticipation of, behaviorally relevant events. The authors draw a distinction between purely internal events, such as anticipation of a stimulus, and external events such as the presentation of a stimulus requiring a motor response, and they suggest that, in their task, firing rate modulation occurs only for behaviorally external events, while internal events may be coordinated in terms of a temporal code. Such differences indicate that the brain may use different forms of coordination to accomplish different tasks. We wish to point out that basic forms of coordination such as bistability or monostability may emerge in both coding schemes, yet in both cases shall appear in forms of transient synchronization. In the case of a temporally coded phase flow, like those shown in Fig. 6, the emergent dynamics of, for example, monostability are such that synchronization does not occur until the system is triggered, resulting in a rapid synchronization followed by a rapid desynchronization.

At the macroscopic level of the brain, lines of research have developed that coincide theoretically with the results presented here. In particular, the theory of neurocognitive networks [35]–[37] argues that cognitive function arises out of the dynamical interaction among the sets of relations between brain regions, and this approach is receiving more and more support [38]. When humans and primates are engaged in a cognitive or behavioral task, and the resulting neural signals are analyzed with measures of causal interaction, the result shows that different functions recruit different networks of brain regions, but that these functions often share one or more active regions. The contribution of a particular region thus depends on the activity of the other active regions with which it is connected. The simulation presented in this study in Fig. 7 highlights exactly this capacity of networks to generate different forms of dynamics. It also suggests that a finer grained mapping of behavioral processes to cortical regions would be possible by looking for the transitions between subspaces of network dynamics, as we have shown could be the case for behavioral or cognitive processes that involve sequences of dynamics.

## Methods

### Excitator dynamics

As an illustrative example of a functionally meaningful process in behavioral neuroscience, we examine the production of simple movements, where it has been suggested that multistability, limit cycle and monostability form a fundamental set of classes of behaviors [5], [39], [40]. Jirsa and Kelso developed a model of the phenomenology of discrete or monostable (the “active transient mode” in [41]) movements in [39]. They developed the model called the Excitator on the principle that 1. there exists at least one stable fixed point in phase space, which represents the equilibrium state of the end effector and 2. a separatrix exists near the fixed point such that large excursions may occur if the system moves far enough away. Such a phenomenology was confirmed in the case of “false starts” by [7]. From [39] Eq 7, we restate a basic two dimensional system satisfying such constraints, illustrated in Fig. S2,
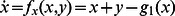


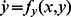
where 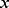 is the position of the end effector and 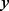 is its velocity. Phase planar models such as the Excitator are common in neuroscience modeling and include for instance the FitzHugh-Nagumo [42], [43] neuron model, which also belongs to the class of excitable systems. In the following, we will show how such excitable dynamics may arise in neural networks. We note that both the effector system (muscles, skeletons, etc.) and the neural networks underlying the production of such excitable dynamics are high-dimensional, while the produced behavioral dynamics are low-dimensional. It is in this general context that the following methods are developed, i.e. to capture the emergence of multiple timescales of dynamical processes from spiking neuron networks.

### Neuron dynamics

We derive different possible implementations of the basic dynamics discussed above in terms of the firing rate and spike timing patterns, and connectivity. We begin with a spiking state variable 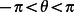, in the style of previous phase oscillators [44]–[46]

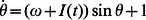
(11)where 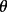 increases as the neuron depolarizes, and at a value of 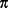, we consider a spike has been generated and the phase is reset to 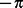, and 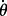 signifies 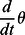; 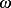 is a parameter prescribing the intrinsic frequency of the oscillator, and 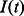 is the non-autonomous external input to this oscillator. The period of oscillation is, determined by integrating Eq 11 from 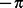 to 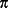, 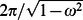. When the radicand is negative, we adopt the convention that the value of the square root be zero, such that the period is infinite and the frequency zero.

Such phase models are obtained using standard techniques in nonlinear oscillator theory [47]. To obtain Eq 11, we use a simple form of excitable dynamics given by
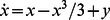


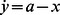
and project to polar coordinates to obtain an averaged radius of
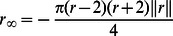
using only the positive finite solution, we obtain the following phase equation
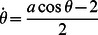
after dropping the higher order periodic terms. We let 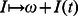 and rotate the system by 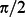 for mathematical convenience to obtain Eq 11. We have assumed that both the input to the neuron and 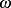 are small and change slowly. As such, the model no longer exhibits a Hopf bifurcation or subthreshold oscillations. Roy et al [48] have shown how such a reduction can be performed with a careful treatment of the bifurcation structure.

In Fig. S3, we show the behavior of this model. We also introduce a dynamics of the intrinsic frequency as a second dimension of this model 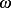, which is biophysically interpreted as a synaptic current but will later serve as a proxy for firing rate [49], [50]:
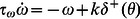
(12)where we define 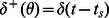 where 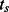 is the time of a spike defined by 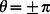 and 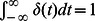. Parameters 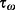 and 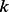 determine the rise and fall timescales.

### Network connectivity

Putting the neuron in a network context, we introduce an additional term in the coefficient of 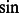 in Eq 11, a weighted sum over the presynaptic neurons, and we add appropriate indices
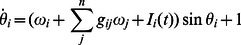
(13)

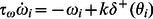
(14)where 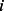 and 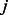 index the post and presynaptic neurons. Each neuron has one free time varying parameter, 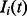, which in the results we will consider as the non-autonomous neuronal input. The network parameters consist of the connectivity matrix 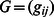, which we will set by hand to achieve specific dynamical attractor structures, and 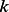 and 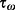 are timescale constants, which separate the dynamics of a neuron's spike and its rate, but in the following, we typically set these values to 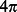 and 20 ms respectively in order to simplify the equations and parameter space.

### Rate reduction

In order to analyze the rate dynamics of Eq 2, we will perform a reduction of the full system to just rate. To this end, we assume that the dynamics of 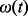 evolves sufficiently slowly, i.e. much slower than the dynamics of 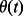, 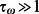. This allows us to approximate the effect of the incoming spike train in Eq 2 via its mean firing rate as given by explicit integration of Eq 11, yielding
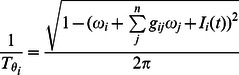
The error of this approximation is shown in Fig. S4. The 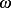 dynamics can now be written as
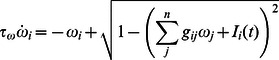
(15)with the following being rewritten for simplicity: 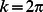 and 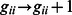.

### Phase reduction

We will also consider a reduction of the network relevant to short timescales based on the assumption 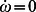 and 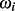 constant, except for the perturbations due to presynaptic spikes which we treat as delta functions, in Eq 11:
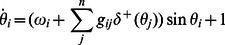
(16)The behavior of such networks can be further analyzed in terms of their phase response curves (also called interaction functions) [51]–[54], which track the change of phase of oscillation in response to an input. The shape of such curves is important as it can directly determine the stable and unstable attractors of the network, allow one to derive or design a networks' firing patterns, to a high complexity [55]. Fig. S5, top panel, illustrates the phase response of an oscillator.

#### Phase locked attractors

To derive the phase locking attractors in a network, we use the set of conditions that for any pair of neurons 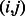, 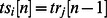, where 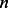 is the index of the spike. Using 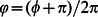, we rewrite these conditions as
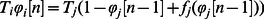
(17)

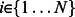
, 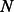 such equations are necessary to uniquely determine values of 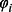 as phase locked states. The stability of such states can be determined by perturbing the steady state and linearizing the map describing whether the perturbation grows or shrinks exponentially, with the approximations 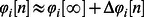 and 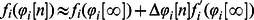 where 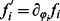. As an example of using this general formulation to derive phase locking attractors, we consider two reciprocally coupled neurons with equal coupling strengths and periods: the two relevant conditions are
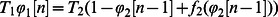
where 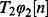 can be obtained by swapping indices. We then introduce the perturbation and subtract the steady state
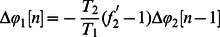
where again the expression for 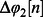 is again found by swapping indices; we then substitute to obtain
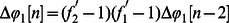
where a fixed point is stable if the eigenvalue 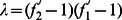 satisfies 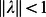, a result general with respect to the nature of 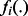 that has been derived before [53].

In general, the existence conditions yield simple stability results whose structure does not depend on the details of the phase response curve. In order to apply such results to Eq 16, we need to obtain its phase response. We note that while the variables 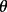 and 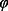 refer to the phase of oscillation, 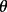 is nonlinear in time, and increases from 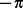 to 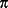, while 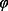, by definition, is linear in time, increasing from 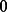 to 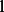.

In order to obtain a phase response curve, we start with the definition of the perturbed period 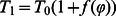, and thus 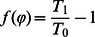, where 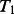 is obtained as a sum of 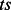 and 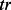, defined by integration of the phase equation
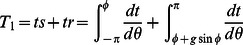
where 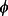 is the value of 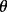 when a presynaptic spike occurs, and 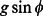 is the instantaneous change in 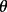 due to the presynaptic spike. Computing the integrals yields 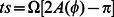, 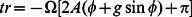, 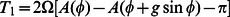, and
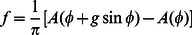
(18)where 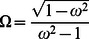 and 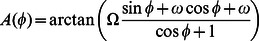 (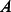 and 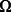 are arbitrary expressions, not amplitude and frequency). Together with specific existence and stability conditions, these expression allow one to determine spike timing patterns produced by Eq 16, and under the adiabatic elimination assumption, those predictions will apply as well to Eq 2, where the period of oscillator is dynamic.

## Supporting Information

Figure S1 **3D network dynamics:** Analogously with Figure 3, the four main Excitator phase flows are generated by a three dimensional network. In each quadrant of this figure, the upper left panel shows a projection of 3D phase space and three simulations, the lower left panel shows the connectivity matrix and the set of three panels on the right show time series corresponding to the three simulations shown in the phase space projection. Black or colored lines are the simulations of the rate equations while the gray jagged lines are from the full spiking network. The paramers for connectivity for the different regimes shown here are taken directly from those used in the bottom panel of 4. **A** fixed point, **B** limit cycle, **C** bistability, and **D** monostability.
(TIFF)

Figure S2 **Excitator flows:** The behavior of an Excitator system is shown here for bistable, limit cycle and monostable dynamics (**A**, **B**, **C**) in the phase space (top) and the time series (bottom). Red lines in the phase space are the nullclines of the system, while black lines show how the phase flows with time on example trajectories.
(TIFF)

Figure S3
**Theta neuron dynamics:** **A** Firing rate as a function of input for the theta neuron described in the text. Two circles give the input and rate for the two sample simulations shown on the right. **B, C** Time series from simulations of 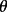 (dashed) and 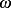 (solid) with two different values of 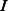.(TIFF)

Figure S4 **Rate reduction approximation** The assumption of the rate reduction in the text is that the mean firing rate captures the relevant information in a spike train. Here we show in **A** and **B**, respectively, cases of low and high firing rates. Upper panels show in blue, red and green curves the omega dynamics time series under a mean firing rate, equal interspike interval (ISI) spike train and Poissonian spike train. Bottom plots show the log sum squared error of the mean firing rate time series with respect to that of equal ISI and Poissonian spike trains in green and blue.
(TIFF)

Figure S5
**Phase response curves:** **A** The phase response curve is found by perturbing the post synaptic neuron with a single presynaptic spike which produces a jump in the phase of the post synaptic oscillator. The black traces show the pre and post synaptic neurons 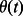 while the gray trace shows the behavior of post synaptic oscillator in the absence of the pre synaptic spike. Phase response curves 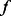 as a function of 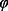 are shown here in **B** and **C** corresponding to positive and negative coupling values, respectively. Both are bimodal, however for both the stronger knee reflects the sign of coupling. The gray lines show the same response curve assuming the phase is linear with time.(TIFF)
