# Supplementary figures and images for: Emergent Dynamics from Spiking Neuron Networks through Symmetry Breaking of Connectivity
Source: PLoS One. 2013 May 17;8(5):e64339. doi: 10.1371/journal.pone.0064339 (PMC3656844; doi:10.1371/journal.pone.0064339)

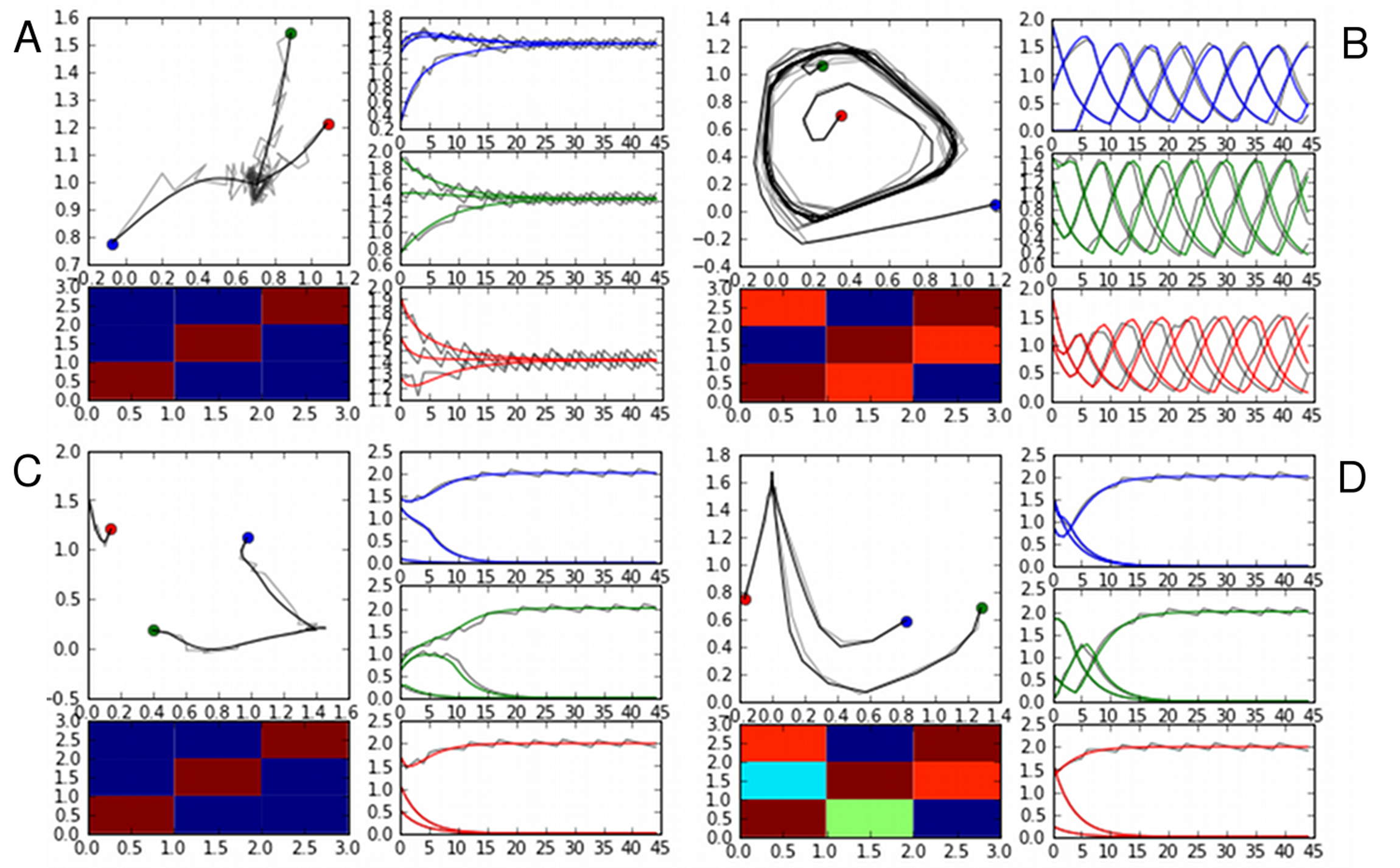

Supplement: Figure S1 — 3D network dynamics: Analogously with Figure 3, the four main Excitator phase flows are generated by a three dimensional network. In each quadrant of this figure, the upper left panel shows a projection of 3D phase space and three simulations, the lower left panel shows the connectivity matrix and the set of three panels on the right show time series corresponding to the three simulations shown in the phase space projection. Black or colored lines are the simulations of the rate equations while the gray jagged lines are from the full spiking network. The paramers for connectivity for the different regimes shown here are taken directly from those used in the bottom panel of 4. A fixed point, B limit cycle, C bistability, and D monostability. (TIFF) [file pone.0064339.s001.tiff]

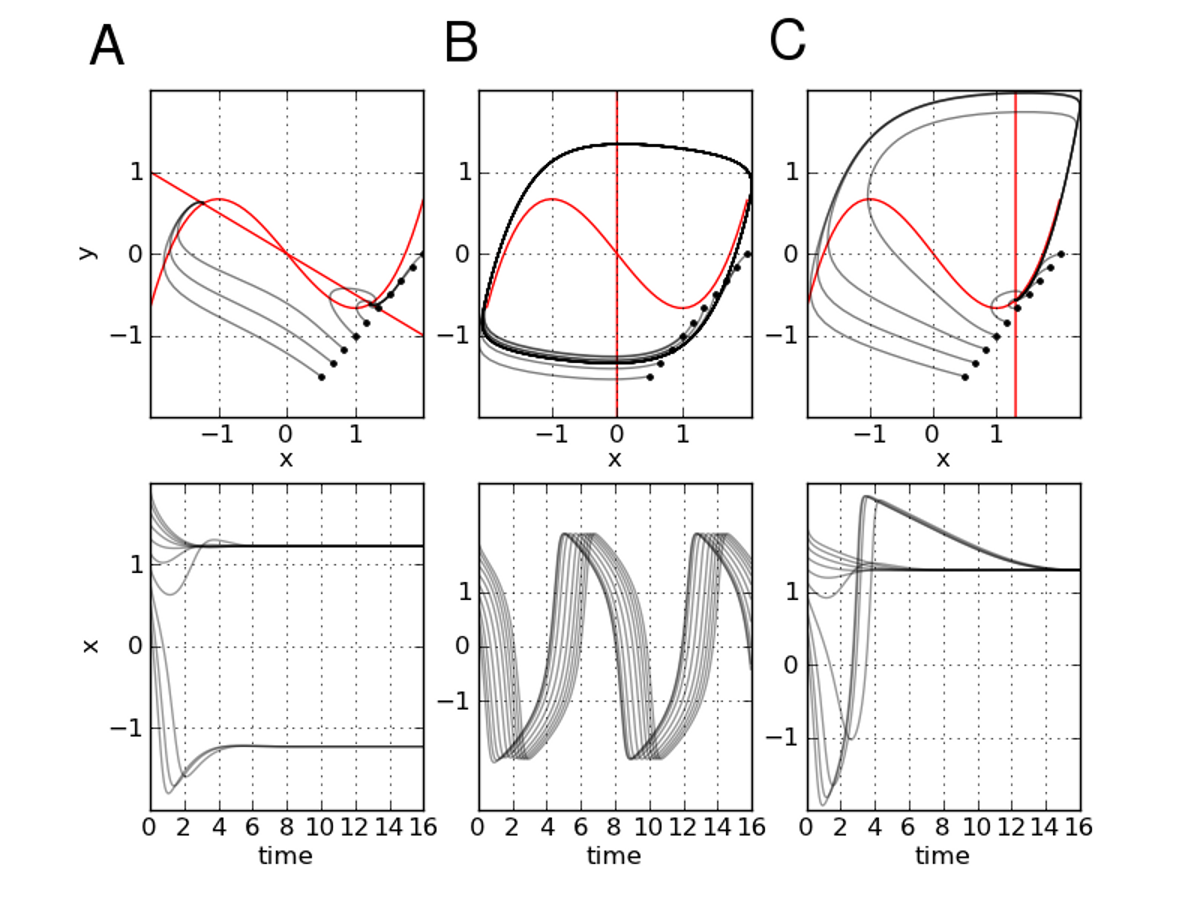

Supplement: Figure S2 — Excitator flows: The behavior of an Excitator system is shown here for bistable, limit cycle and monostable dynamics (A, B, C) in the phase space (top) and the time series (bottom). Red lines in the phase space are the nullclines of the system, while black lines show how the phase flows with time on example trajectories. (TIFF) [file pone.0064339.s002.tiff]

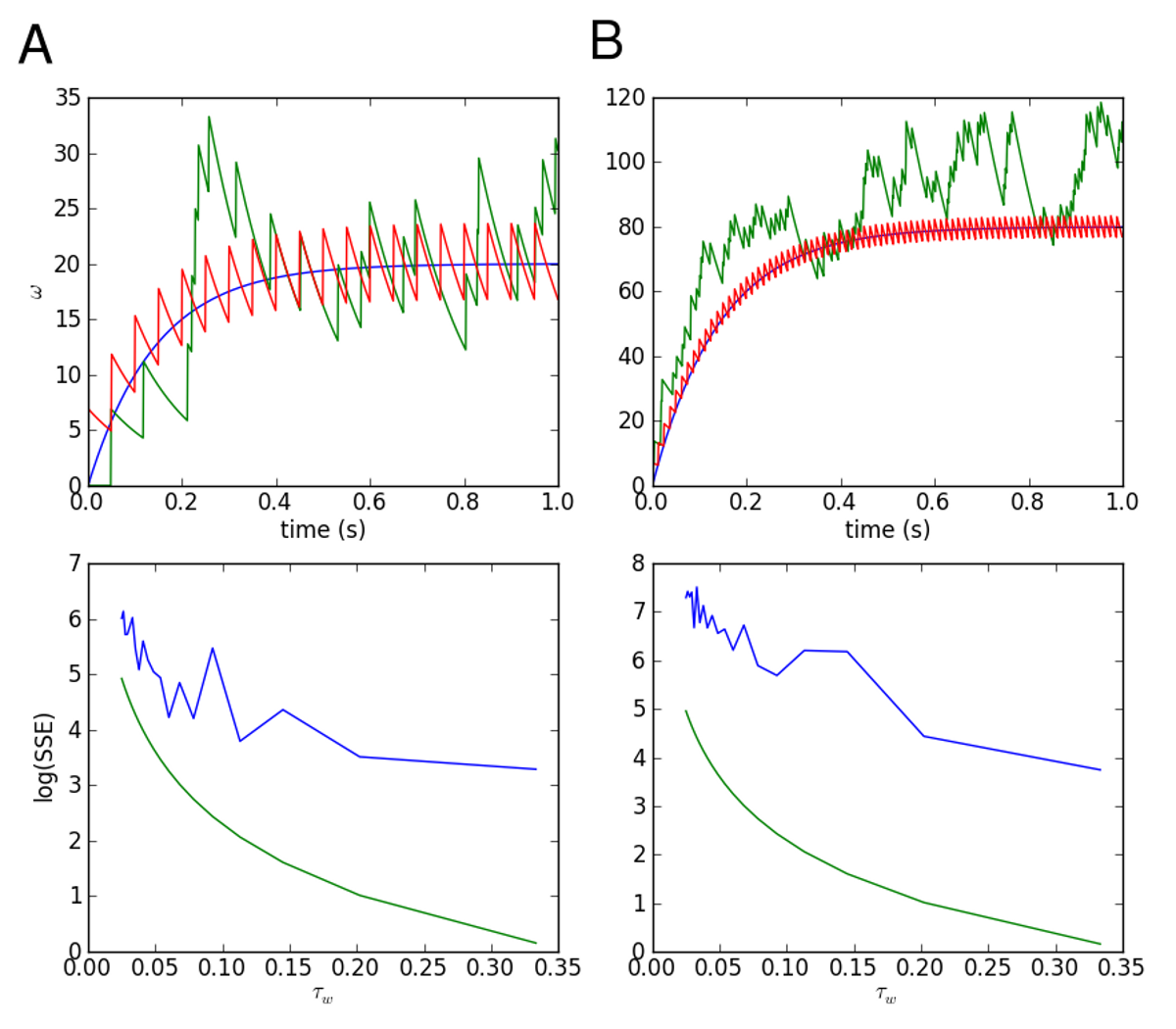

Supplement: Figure S4 — Rate reduction approximation The assumption of the rate reduction in the text is that the mean firing rate captures the relevant information in a spike train. Here we show in A and B, respectively, cases of low and high firing rates. Upper panels show in blue, red and green curves the omega dynamics time series under a mean firing rate, equal interspike interval (ISI) spike train and Poissonian spike train. Bottom plots show the log sum squared error of the mean firing rate time series with respect to that of equal ISI and Poissonian spike trains in green and blue. (TIFF) [file pone.0064339.s004.tiff]
